# Snowpack-stored atmospheric surface-active contaminants traced with snowmelt water surface film rheology

**DOI:** 10.1007/s11356-020-10874-1

**Published:** 2020-09-23

**Authors:** Stanisław J. Pogorzelski, Paweł Rochowski, Maciej Grzegorczyk, Katarzyna Boniewicz-Szmyt

**Affiliations:** 1 Institute of Experimental Physics, Faculty of Mathematics, Physics and Informatics, University of Gdańsk, Wita Stwosza 57, 80-308 Gdansk, Poland; 2 MGE, Lipowa 7, 82-103 Stegna, Poland; 3 Department of Physics, Gdynia Maritime University, Morska 81-87, 81-225 Gdynia, Poland

**Keywords:** Snowmelt water, Atmospheric contamination, Surface-active organics, Surface film rheology, Snowpack morphology, Surface viscoelasticity

## Abstract

The aim of the study was to quantify the adsorptive and thermo-elastic properties of snowmelt water surface films and their spatial-temporal evolution with snowpack structure characteristics and the entrapped surface-active organic composition. Surface pressure–area (*π*-*A*)_*T*_ isotherms, surface pressure-temperature (*π*-*T*)_*A*_ isochors, and stress–relaxation (*π*-*t*) measurements were performed using a Langmuir trough system on snowmelt water samples collected in a large-scale field studies performed at several industrialized and rural Tricity (Gdansk, Poland) areas at various environmental conditions and subsequent stages of the snowpack melting progress. Since the snow-melted water composition and concentrations of surface active organic matter fractions therein are largely undetermined, the force-area isotherm scaling formalisms (2D virial equation and 2D film scaling theory of polymeric films) were adapted to the complex mixture of surfactants. The surface film parameters and their spatial and temporal evolution turned out to be unequivocally related to principal signatures of the film-forming materials: surfactant concentrations (*π*, *A*_lim_), surface activity (*E*_isoth_, |E|), film material solubility (*R*), surface material miscibility and 2D architecture complexity (*y*, *β*_*s*_), molecular thermal mobility (*π*_*k*_), and a timescale of the relaxation processes within the film (*τ*_*i*_, |*E*|). Moreover, the parameters appeared to be correlated with snowpack structure characteristics (snow density *ρ*, specific snow area SSA, snow cover thickness), sample age time, and anthropogenic atmospheric contamination pressure source locations. In particular, *E*_isoth_ was found to be related to *ρ* and SSA, while *R* correlated with the solubility of film-forming organics which turned out to be long-chain fatty acids; similarly, spatial profiles of *E*_isoth_ revealed the peak values next to the areas being under a severe anthropogenic air pollution pressure. Snowmelt water films stand for a structurally heterogeneous (*y* > 10) interfacial system where several transition processes of differentiated time-scales (relaxation times from 7 to 63 s) took place leading to the apparent surface viscoelasticity. To sum up, the established surface rheological parameters could serve as novel indicators, based solely on physical attributes, allowing to follow the snowpack evolution, and its melting polymorphism in order to test or improve the existing snow-entrapped organics release models based on chemical analyses. The cross-correlation functional dependences of practical value remain to be established on the larger data set.

## Introduction

It is concluded that snow influences the atmospheric hydrologic cycles of organic materials. Snow and ice melting processes affect atmospheric contamination load to marine and terrestrial systems. Snow appears to be an effective collecting and temporary storage medium of organic chemicals from the atmospheric environment. Falling snow, after trapping contaminants, concentrates them in a snowpack volume. Airborne contaminants deposited by snow fall come from washing out the aerosol and sorbing the vapor phase (Franz 1994). As a result of snow metamorphosis and melt processes, the contamination is concentrated in a snowpack and further snowmelt released. The problems to be addresses are as follows: what are chemical hydrophilic–lipophilic balance (HLB) of the entrapped material in the light of the snowpack morphology and to which extent the physical conditions undergoing during the melting time affect these phenomena? . A large specific area of a snowpack allows to adsorb hydrophobic organic compounds to a great extent (Wania et al. 1998). It is supposed that more hydrophilic part of the organic contaminants mixture (of less surface activity) is released in an early stage of melting, although the hydrophobic fraction attached to dry aerosol particles is discharged in the final melting process stages (Meyer and Wania 2008). Urban stormwater and snowmelt pollution contributes significantly to the deterioration of surface waters quality in many locations. Consequently, the sources of such pollution have been studied for the past 50 years, with the vehicular transportation sector and the atmospheric deposition identified early as the major pollution sources (Müller et al. 2020). Since the snowpack contamination content appears to vary in a wide range, Herbert et al. (2006) considered different sampling methods to improve the level of precision for following the organics concentration routes, fluctuating atmospheric concentrations influencing interface adsorptive organics exchange kinetics, and varying the snowpack physical properties (Kinar and Pomeroy 2015). Snowpack signatures and its evolution were topics addressed in several theoretical and experimental works.

The snowpack plays an important role in providing a storage medium for atmospherically transported pollutants. Snowpack is a unique indicator in assessing both local and transboundary contaminants. Snow on an open landscape on a hill is most susceptible to airborne pollution (sulfates, copper, nickel), whereas city snow is most affected by local pollutants (turbidity, lead; Dinu et al. 2020).

There are no any efficient enough theories describing in detail the rate of chemicals spatial and temporal concentration evolution, and physical processes at the interface between the atmosphere and the underlying snow, controlling the exchange of energy and mass (Kinar and Pomeroy 2015). The application of numerical modeling of the snowpack provides information otherwise unavailable on the present and future state of the snowpack and its mechanical stability (Morina et al. 2020).

Chemicals identified in snowpack samples may include a contribution of both gaseous and aerosols dry deposition forms. According to Garbarino et al. (2002) and Herbert et al. (2005), organic chemical contaminants present in snow include persistent organic pollutants (POPs) such as polychlorinated biphenyls (PCBs), and organochlorine (OC) pesticides, polycyclic aromatic hydrocarbons (PAHs), n-alkanes, and phthalates. To quantify snow scavenging, the total ratio of organic contaminant concentration in snowmelt water and the atmosphere known as the interfacial partition coefficients is evaluated. It is clear that snowpack morphology depends on several ambient and time conditions, undergoing many physical changes (Wever et al. 2014). Snowpack properties are followed by snow metamorphosis brought about by changes in wind speed, temperature, relative humidity, and solar radiation. During an aging, the snowpack metamorphosis and melting evolve. There are a number of processes that simultaneously take such as repartitioning and translocation within snowpack, volatilization, and drainage with melt water. The higher heterogeneity of snowpack structure is observed mainly due to differences of snow accumulation types identified by grain shape and size characterization (Colbeck 1991). Grain size is classified from “very fine” (< 0.2 mm) to “very coarse” (2.0–5.0 mm). Other of the most important physical parameters are density (*ρ*), and specific surface area (SSA) of a snow layer (Raleigh and Small 2017). Temporal transition of snow leads to a progressive decrease in SSA and snow porosity accompanied by an increase in *ρ* (Herbert et al. 2006). It depends on the age and “weathering” of the snow; SSA generally falls between 100 and 1500 cm^2^ g^−1^ (Domine et al. 2002). Values of *ρ* are ranging in between 0.01 ÷ 0.1 kg L^−1^ with a porosity of about 95%, for fresh snow, and increase with the subsequent snow metamorphosis to 0.3 ÷ 0.5 kg L^−1^ with a porosity of 50% (old snow; Herbert et al. 2006). Snowpack is a variable medium with physical properties that can be changed by fluctuating climate conditions, affecting both *ρ* and SSA (Wurzer et al. 2016). As with snow density, snow temperatures are rarely of primary interest in snow studies. However, a correct representation of the temperature profile of the snowpack is required, as it has a large influence on the snow metamorphism (grain shape and size) and settling rates. Temperature gradients drive moisture transport and have a strong influence on the grain growth. Furthermore, temperature profiles are an indicator of whether the combination of the surface energy balance, the ground heat flux, and the internal heat conductivity of the snowpack is adequately approximated (Wever et al. 2015).

A certain role in snowmelt water film formation is played by microorganisms capable of producing surface active organics. Microbial abundance in snowpack generally ranges from 10^3^ to 10^4^ cells per ml of melted snow. Within the snowpack, microbes are subjected to a range of physico-chemical conditions related to the specific structure and composition of snow. When snow forms, it scavenges organic contaminants and persistent pollutants from anthropogenic sources that might act as carbon sources for snow microbial communities. Previous measurements of dissolved organic carbon (DOC) in non-saline snow over sea ice in the high Arctic have indicated that DOC concentrations are much lower in bulk melted snow (10X less) than in underlying sea ice brine (37.5 mg C L^−1^), where nutrients and microbial cells are concentrated within the ice matrix (Maccario et al. 2019). The occurrence of *Cyanobacteria*, likely blooming in patches in surface snow, and of diatoms in saline snow, as well as some non-oxygenic producers, highlighted the potential for primary production. Basic information on surfactants and overview of pollution of different ecosystems caused by them (i.e., presence, behavior in the environment, surface activity and classification) can be found in Olkowska et al. (2014).

Moreover, solar irradiation-driven photochemical reactions at the snow surface may lead to variability of organics composition and concentration, and further forming interfacial films exhibiting unique adsorptive and thermoelastic signatures (Boniewicz-Szmyt and Pogorzelski 2016). The surface rheology quantification of the snowmelt surface films with solely physical parameters was proposed in this study, for the first time. It has to be pointed out that no chemical analyses and identification of organics components in the snowpack samples were performed. A variety of the established parameters in relation to the environmental factors (film temperature, ionic strength and pH of the aqueous subphase, sampling location, wind speed, timescale of relaxation processes taking place in a multicomponent natural film) at the natural seawater interfaces have been already discussed in detail elsewhere (Mazurek et al. 2008; Pogorzelski and Kogut 2003). A set of the surface film structure and its rheology parameters allowed us to quantify several multicomponent film signatures like the film material composition, its compounds interfacial miscibility, molecular architecture and thermal molecular mobility, 2D thermodynamics, and surface dilatational viscoelasticity. It is assumed that the fate of organic surface-active contaminants during the melting of a snowpack can be followed with these physical indexes similarly as it was already performed to quantify the rain water film characteristics (Mazurek et al. 2006).

The proposed approach can be useful in the atmospheric organic matter dynamics tracing, as already established formalisms in the atmospheric studies based on the acids concentration ratio or the fractional acidity describing the material dissolved in bulk samples of hydrometeors. It is supposed that snow is more effective in scavenging airborne chemicals than rain (Wania 1997; Wania et al. 1998). Moreover, the cross correlations between the surface film rheology and structural snowpack morphology (snow density, aging time, specific surface area) allow one to create a promising practical tool for the atmospheric contamination pathways tracing, providing comprehensive information apart from chemical data analyses. The aim of the large-scale field studies, performed at surroundings of University of Gdansk Campus at Gdansk-Oliwa, Poland, was to trace structure snowpack evolution and spatial air-born organics pollution concentrations with surface signatures of snowmelt water films.

Recently, a new film structure quantification formalism was developed (Boniewicz-Szmyt and Pogorzelski 2018b). In brief, the multidimensional vector which coordinates stand for the normalized film parameters can be created as the film unique structural state signature. The structural similarity of the considered films can be expressed as a Cartesian distance between two vectors of the analyzed film and the standard one from the reference data base, respectively.

## Methodology

### Film structural parameters: the scaling procedures

The intensity of the film-effect depends strongly on film surface concentration, composition, and viscoelastic properties of snowmelt water surface films. A thorough structural and compositional picture of surface active fraction snowpack-collected contaminants cannot be created. Of particular interest are the relatively high levels of the hydrophobic organic fraction components occurring in the snow cover, which play a role of the so-called end-members affecting the film structure to a great extent. Wania (1997) has introduced the model which provides a first quantitative treatment of the processes affecting hydrophobic organic contaminants fate in an aging homogenous snowpack. The performed calculations introduce assumptions on the signatures in physical snowpack properties, especially snowpack specific surface area SSA and its density.

In order to derive the snowmelt surface film adsorptive and thermo-elastic significant parameters, surface pressure-area isotherms (*π*-*A*)_*T*_, surface pressure–temperature isochors (*π*-*T*)_*A*_, and surface pressure–stress (*π*-*t*) dependences were analyzed where chemical identification of film forming compounds was avoided. The multicomponent film morphology is quantified with physicochemical composition quantities (Γ, *A*_lim_, *M*_*W*_, *E*_isoth_), film entropy and molecules mobility (*β*_*S*_, *π*_*k*_) attributes, film-forming material self-solubility (*R*), interfacial architecture complexity (*y*), and dilatational viscoelasticity (|*E*|, *τ*).

The surface film isotherm (*π*-*A*)_*T*_ can be described by the 2D analog of the ideal gas law (Adamson and Gast 1997):

1 $$ \pi {A}_m= kT, $$

where *k* is the Boltzmann constant, *A*_*m*_ is the area per film molecule related to Gibbs’ adsorption Γ, *A*_*m*_ = 1/ Γ*N*_*A*_, *N*_*A*_ is the Avogadro number, and *T* is the temperature in Kelvin.

The surface pressure of the film π is defined as

2 $$ \pi ={\gamma}_0-\gamma, $$

where *γ*_0_ is the surface tension of the solvent (water) and *γ* is surface tension of surfactant solution.

Natural multicomponent surfactant film isotherm does not follow the ideal gas behavior. In order to derive the film structural parameters, the mean number of moles *n*_*m*_ present in the film, specific limiting area *A*_lim_, and mean molecular mass *M*_*W*_ of the film-forming material, the 2D virial equation of state was introduced (Barger and Means 1985):

3 $$ \pi A={C}_0+{C}_1\pi +{C}_2{\pi}^2, $$

where *C*_0_, *C*_1_, and *C*_2_ are the virial coefficients and *A* is the film-covered area (in cm^2^), which after adaptation of the best-fit procedure to the registered (*π*-*A*)_*T*_ isotherms, allowed the said parameters to be determined, as already described in (Pogorzelski and Kogut 2003).

The film classification is based on the 2D film state exhibited. The film state evolution undergoes the following stages as follows: from gaseous (*G*) → liquid expanded (LE) → liquid condensed (LC) → solid (*S*). The particular state is attributed to the taken dilatational surface elasticity modulus *E*_isoth_ (or Gibb’s modulus), which reflects the static response (Δπ) of a film to the applied film area change (Δ*A*), for the film being at its thermodynamic equilibrium, defined as (Adamson and Gast 1997):

4 $$ {E}_{\mathrm{isoth}}=-A{\left(\raisebox{1ex}{$\partial \pi $}\!\left/ \!\raisebox{-1ex}{$\partial A$}\right.\right)}_T, $$

where the initial part (0 < π < 1 mN m^−1^) of the isotherm is considered.

The isotherm hysteresis, observed during the film area compression–expansion cycle, results from various molecules configurations undergoing in the film Mazurek et al. 2008), related to the structural entropy change Δ*S*_*c*_ (Δ*S*_*c*_ = Δ*W*/T). The work difference between compression and dilation of the film surface: Δ*W* = *W*_com_ − W_dil_ results from the integration of isotherm plots between the initial *A*_*i*_ and final *A*_*f*_ film areas). The surface compression work (and expansion as well) is defined (Pogorzelski and Kogut 2003):

5 $$ {W}_{\mathrm{com}}={\int}_{A_i}^{A_f}\pi dA $$

The isotherm hysteresis-related quantity, i.e., isotherm reversibility *R*:

6 $$ R=\left(\raisebox{1ex}{${W}_{\mathrm{dil}}$}\!\left/ \!\raisebox{-1ex}{${W}_{\mathrm{com}}$}\right.\right)\cdotp 100\% $$

allowed the entropy effect quantification in films differentiated in their chemical composition, related to the film-forming material solubility (Pogorzelski and Kogut 2003).

The miscibility and self-organization of film-composing surfactants within the interfacial structure can be foreseen by adopting the 2D polymer film scaling approach. The scaling exponent *y* can be obtained from the dependence *E*_isoth_ = *yπ*, under the high-frequency film compression condition, where the film material is assumed to be effectively insoluble (Pogorzelski 1996). The taken particular *y* values point to a certain surfactants structure morphology as follows: values of *y* lower than 3.5 represent an uniformly spatially mixed film (so-called “good “solvent condition), for *y* ~ 8 (“theta solvent condition”) film heterogeneity as surface aggregates or 2D micelles of film compounds is expected to appear, and finally largest *y* values (> 10–16; “poor” solvent condition) correspond to a horizontally layered surfactants structure with the most surface-active (and insoluble) component residing on the top of such an interfacial sandwich-like texture.

### Thermal and viscoelastic film parameters

Analyses of the surface pressure-temperature isochore (*π*-*t*)_*A*_ exhibit 2D phase transitions which are not revealed in isotherms (*π*-*A*)_*T*_. That allows the range of critical phenomena in the film to be specified like: film collapse or phase transitions of higher orders (Rosenholm et al. 2003). The surface pressure-temperature coefficient is defined as (Adamson and Gast 1997)

7 $$ {\beta}_S={\left(\raisebox{1ex}{$\partial \pi $}\!\left/ \!\raisebox{-1ex}{$\partial T$}\right.\right)}_A. $$

From Eq. (2) we have

7a $$ {\beta}_S={\left(\raisebox{1ex}{$\partial {\gamma}_0$}\!\left/ \!\raisebox{-1ex}{$\partial T$}\right.\right)}_A-{\left(\raisebox{1ex}{$\partial \gamma $}\!\left/ \!\raisebox{-1ex}{$\partial T$}\right.\right)}_A. $$

The parameter *β*_*s*_ is a difference between the surface entropy of water and the surfactant solution (Adamson and Gast 1997) and stands for a direct measure of the interfacial film structure complexity.

*β*_*s*_ is obtained from a slope of the straight line tangent to the experimental plot computed using a least-squares fitting procedure and applied to the particular temperature ranges below and above the cusp points evidenced at each isochore plots (see Fig. 3 in Mazurek et al. 2006, for example).

A measure of the kinetic (thermal) mobility of film-forming molecules is so-called kinetic surface pressure π_k_ (Harkins 1952):

8 $$ {\pi}_k={\beta}_ST. $$

In general, the apparent film pressure consisting of three components is given by (Rosenholm i in., 2003)

9 $$ \pi ={\pi}_k+{\pi}_{\mathrm{coh}}+{\pi}_r, $$

where π_coh_ is cohesive pressure corresponding to the van der Waals attractive forces between the hydrocarbon chains and π_r_ is pressure resulting from the electrostatic repulsion forces between charged head groups of monolayers (Gong et al. 2002), which is assumed to be negligible for non-ionic surfactant layers. As it was found empirically, for long-chain surfactants films, *π*_coh_ is related to the number of methylene groups in the hydrocarbon chain and molecular area being affected by the subphase pH.

The surface film dilational viscoelasticity results from any molecular relaxation process (bulk solution to surface diffusion, partial film collapse, surfactant molecule separation, and molecular structure reorganization) taking place in interfacial film region. The surface dilational viscoelastic modulus *E* becomes a complex quantity with real *E*_*d*_ (called dilational elasticity and imaginary *E*_*i*_ (related to the dilational surface viscosity *E*_*i*_ = *ωη*_*d*_) components. Assuming the periodic films are oscillations with the angular frequency *ω*, *E* modulus can be expressed as *E* = *E*_*d*_ + *iE*_*i*_ = *E*_0_cos *φ* + *iE*_0_sin *φ*, where *φ* is the loss angle (with tan *φ* = *E*_*i*_/*E*_*d*_), and *E*_0_ = − *Δπ*/(Δ*A*/*A*) is the amplitude ratio: surface pressure stress to area strain (Ravera et al. 2005).

As already argued in Boniewicz-Szmyt and Pogorzelski (2016), the above surface viscoelasticity determination formalism can be adopted to the surface pressure-stress mode experiment. First, the surface pressure-time (*π*-*t*) response is recorded after a step-wise rapid (Δ*t* = 0.2–1.5 s) film area deformation Δ*A*/*A*_0_ (= 0.07–0.23) and analyzed to obtain the characteristic relaxation times τ_i_ of the transition processes by plotting ln *π*(*t*) vs *t*. The modulus *E* real and imaginary components are given by (Jayalakshmi et al. 1995)

10a $$ {E}_d={E}_0\left[\raisebox{1ex}{$1+\Omega $}\!\left/ \!\raisebox{-1ex}{$1+2\Omega +2{\Omega}^2$}\right.\right], $$

10b $$ {E}_i={E}_0\left[\raisebox{1ex}{$\Omega $}\!\left/ \!\raisebox{-1ex}{$1+2\Omega +2{\Omega}^2$}\right.\right], $$

where:

11 $$ {E}_0=\frac{\left({\pi}_0-{\pi}_{\infty}\right)}{\Delta  A/{A}_0}. $$

|*E*| is the modulus amplitude:

12 $$ \left|E\right|=\sqrt{E_d^2+{E}_i^2}\Big), $$

while:

13a $$ \Omega =\sqrt{\raisebox{1ex}{$\Delta  t$}\!\left/ \!\raisebox{-1ex}{${\tau}_i$}\right.}, $$

13b $$ \mathrm{tan}\upvarphi =\raisebox{1ex}{$\Omega $}\!\left/ \!\raisebox{-1ex}{$1+\Omega $}\right., $$

### Materials, instrumentation, and experimental procedures

Sampling of snowpack probes were performed in rural (Tricity Landscape Park), marine seashore (near Gulf of Gdansk, Baltic Sea), and urban (near traffic-busy street—Grunwaldzka) locations in Gdansk (Poland) during a winter season (January–March, 2016). Sampling methodologies and field measurements usually involved taking a large volume fraction of snow samples using metal grab and collected in a drain at the bottom of the sampling vessel.

The collected snowpack samples were provided to the laboratory, within an hour after the sample collection, for further snowmelt processing, in particular, to perform force-area studies on snowmelt surface films. The conventional Langmuir trough area *A*_0_ (= 1200 cm^2^) was compressed with an average deformation speed *v* = 0.6 cm^−2^ s^−1^ by moving two PTFE sliders toward each other symmetrically around the film pressure sensor. Surface pressures were measured with a Wilhelmy plate technique using a piece of filter paper (Whatman No1, Madstone, England; 5-cm wide) connected to a force sensor (GM2 + UL5, Scaime, France). They were accurate to within 0.1 mN m^−1^. After equilibrating the snowmelt water sample in the trough for 30 min, i.e., the standard period chosen for a practical purpose, (*π*-*A*)_*T*_ isotherms were recorded in the temperature interval 5–35 °C. Samples were heated from the Langmuir trough bottom realized with a water thermostated system (temperature controlled with an accuracy 0.1 °C using a thermocouple) to obtain (*π*-*T*)_*A*_ isochors. Dynamic film characteristics were evaluated from stress–relaxation studies (Boniewicz-Szmyt and Pogorzelski 2016), using a novel frame-shaped Langmuir trough apparatus (see Fig. 2 in (Boniewicz-Szmyt and Pogorzelski 2016)). The surface pressure-time response *π* (*t*) of a film to a step rapid (Δ*t* = 0.19–1.1 s) relative surface area deformation Δ*A*/*A*_0_ (= 0.07–0.23) applied to the sample by barrier movement was registered for several minutes. The reported structural, thermodynamic, and viscoelastic surface parameters stand for an average value over 6–9 measuring runs performed for the given sample. A further detailed description of the measuring procedures and physical conditions adopted in surface pressure-area and dynamic surface pressure measurements can be found in Pogorzelski et al. (2006). The boxes of known volume were all filled to the same level to easily detect the compaction process (SSA) and weighted (with an electrobalance Δ*m* ± 10^−5^ g) to measure the snowpack density *ρ*.

## Results and discussion

### Snowmelt water film surface parameters and snowpack structure evolution

**Fig. 1:**
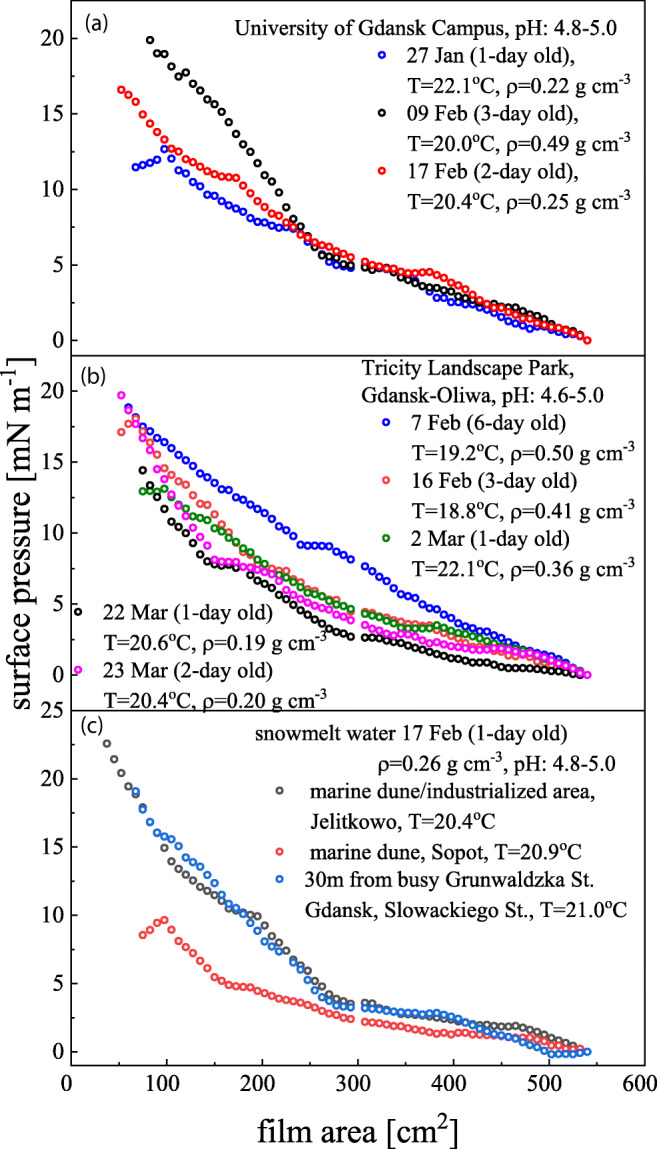
Snowmelt water film isotherms (*π*-*A*)_*T*_ sampled in 2016 at **a** University of Gdansk Campus in Oliwa, **b** Tricity Parku Landscape Park (Gdansk-Oliwa) after 1–6 days from the snowfall event, and **c** beach dune area close to Gulf of Gdansk (Baltic Sea) and busy street (Grunwaldzka in Gdansk) on the same day

The exemplary isotherms of snowmelt water films are shown in Fig. 1a–c. Since Γ = *π*/RT, the obtained higher surface film pressures for longer-aged snowpacks pointed to higher surface active material content while the film composition (and surface activity) remained almost the same (similar *E*_isoth_ = 5.6–7.5 mN m^−1^). A positive correlation can be noticed between the final surface pressure and the snow-cover density. The isotherm shape pointed to a surface film of a G-LE (gas-liquid expanded) type where *E*_isoth_ ~ *π*. Values of Γ were ranging from 0.24 to 0.56 · 10^6^ mol m^−2^ corresponding to *A*_lim_ (89.5–33.5 nm^2^) characteristic for diluted surfactant solutions (Adamson and Gast 1997). Similar signatures of the isotherms and the related parameters were found for surfactants occurring in aerosol samples (Forestieri et al. 2018). Intercomparison of the selected parameters: *E*_isoth_, *y*, *R i* Δ*S*_*c*_ suggested that film-forming surface active materials contained in snowmelt water are less soluble (*R* ↑, Δ*S*_*c*_↓) of lower surface surfactants miscibility (large *y* ↑) than evidenced in rain water samples (Mazurek et al. 2006) but of similar makeup (comparable *E*_isoth_). Such a film evolution to the new molecular arrangements state is an evidence of a certain loss of the system degrees of freedom leads to a larger entropy change than it is supposed for a structure less film. It appeared that *R* is related closely to the solubility of the film-forming material taking higher values for slightly soluble materials (Pogorzelski et al. 2006). For instance, the following structural parameters were derived for the snowfall event (fresh snow) in Oliwa on Feb. 17, 2016: *E*_isoth_ = 26.23 ± 2.39 mN m^−1^; *R* = 78.9 ± 7.8%; Δ*S*_*c*_ = (− 9.91 ± 1.25) × 10^−7^ J K^−1^ and *y* = 15.3 ± 1.4. The isotherm plots exhibited the kink at *π* = 4.8 ÷ 5.0 mN m^−1^ caused by the breakdown of stearic acid, which is forced out of the film at its equilibrium surface pressure (ESP) and the other one at around π = 7.8 ÷ 9.0 mN m^−1^ characteristic for the breakdown of palmitic acid (another fatty acid occurring at high concentrations in atmospheric waters, found in the rain water films as well (Mazurek et al. 2006)). It suggested that stearic and palmitic acid have the highest concentrations among the other surface active organics in rainwater capable of determining the rheology of snow melt water films (Seidl 2000). The isotherms became more steeper (*E*_isoth_ ↑), and laid above the isotherms corresponding to the fresh snow samples as the snow cover is aged (Fig. 1a–b) that pointed to the increased content (concentration) of more surface active compounds. A spatial distribution of organic contaminations in the snowpack can be considered for two their basic classes, i.e., “dry” and “wet,” as argued by Meyer and Wania (2008). The first class represents a fresh snowfall cover with a large specific snow area (SSA) ~ 1000 cm^2^ g^−1^, low density (~ 0.05 g cm^−3^), and a small content of organic matter (OM) ~ (0.009 μg mL^−1^ per snowpack volume). The other one corresponds to the long-aged, musty snow cover of low SSA (~ 125 cm^2^ g^−1^), high density (~ 0.40 g cm^−3^), with significant contents of OM (0.18 μg mL^−1^).

The snowfall covers, studied here, belong to the substrata characteristic for near seashore regions (Meyer and Wania 2008). This kind of snow layer is “warm” and deep (75–500 cm) with low temperature gradients, high density, and small SSA, composed of large dimension snow grains, exhibiting the melting mechanism characteristic for “wet” snow.

The investigated snow cover can be classified as the “wet” one with high density *ρ* = 0.20 (fresh snow) ÷ 0.50 (after 7 days elapsed from the snowfall event) g cm^−3^, and rather low SSA ~ 120 cm^2^ g^−1^. Much higher π values were found in film samples collected in heavily air polluted areas Fig. 1c. It could be an evidence of snowpack enrichment in surface active organic contaminants in areas of the increased dust (dry aerosol) emission (Hitchins et al. 2000). It indirectly confirmed the assumption on the accumulation of slightly soluble fatty acids at the mineral dust particles that progresses with the snowpack cover age and lowering the distance to the dust source (Meyer and Wania 2008). The evidenced film parameter variability goes in line with the model of organic contaminant release during snow melting (Meyer 2008). The sequential release order of a particular organic compounds during the melting process depends on the possessed environmental partitioning ability between atmosphere/liquid/soil ground phases (adsorption effectiveness, solubility, and hydrophobicity), and physical characteristics of snow cover layer (density, SSA, soil properties, vertical temperature gradients). Well water-soluble organic compounds are supposed to be released with significantly increased concentrations in the initial stages of snow melting, whereas a greater majority of slightly soluble (most hydrophobic) materials, likely adsorbed at mineral dust particles, are observed at final stages of the process. Warmer and permeable soil substrata slow down the snowmelt flow and dissolves the contamination. Seasonal maxima of organic contamination concentrations registered in snowmelt water are positively correlated to temporal concentration maxima in atmosphere, soil, and natural water bodies (Gouin et al. 2005).

It is of interest to compare surface rheology of natural organic films formed on marine water, rain water, and snowmelt water all collected and studied in the same locations, neighboring Baltic Sea coastal areas. The isotherm-derived parameters from the fresh snow sampling event at Gdansk on March 6, 2016, are equal: *E*_isoth_ = 28.46 ± 3.41 mN m^−1^, ∆*S*_*c*_ = − 0.69 (± 0.08) × 10^−5^ J m^−2^ K^−1^, *R* = 80.9 ± 9.7%, and *y* = 14.3 ± 1.7, and are comparable to the rain water ones (collected at Gdansk on March 23, 2016): *E*_isoth_ = 27.26 ± 3.27 mN m^−1^, ∆*S*_*c*_ = −0.24 (± 0.02) × 10^−5^ J m^−2^ K^−1^, *R* = 88.3 ± 8.6%, and *y* = 20.7 ± 2.5, and significantly differing, from characteristic for natural marine surface films (at Gdansk-Orlowo on 24 March 2016): *E*_isoth_ = 19.31 ÷ 42.69 mN m^−1^, ∆*S*_*c*_ = − (0.09 ÷ 7.04) × 10^−5^ J m^−2^ K^−1^, *R* = 51.60 ÷ 64.20%, and *y* = 3.3 ÷ 13.9. Comparing rain water and snowmelt water film structural parameters indicated a similar chemical nature of the film components (similar values of *E*_isoth_ and *R*) with more structural compounds segregation degree in the case of rain water materials (higher value of y).

**Fig. 2:**
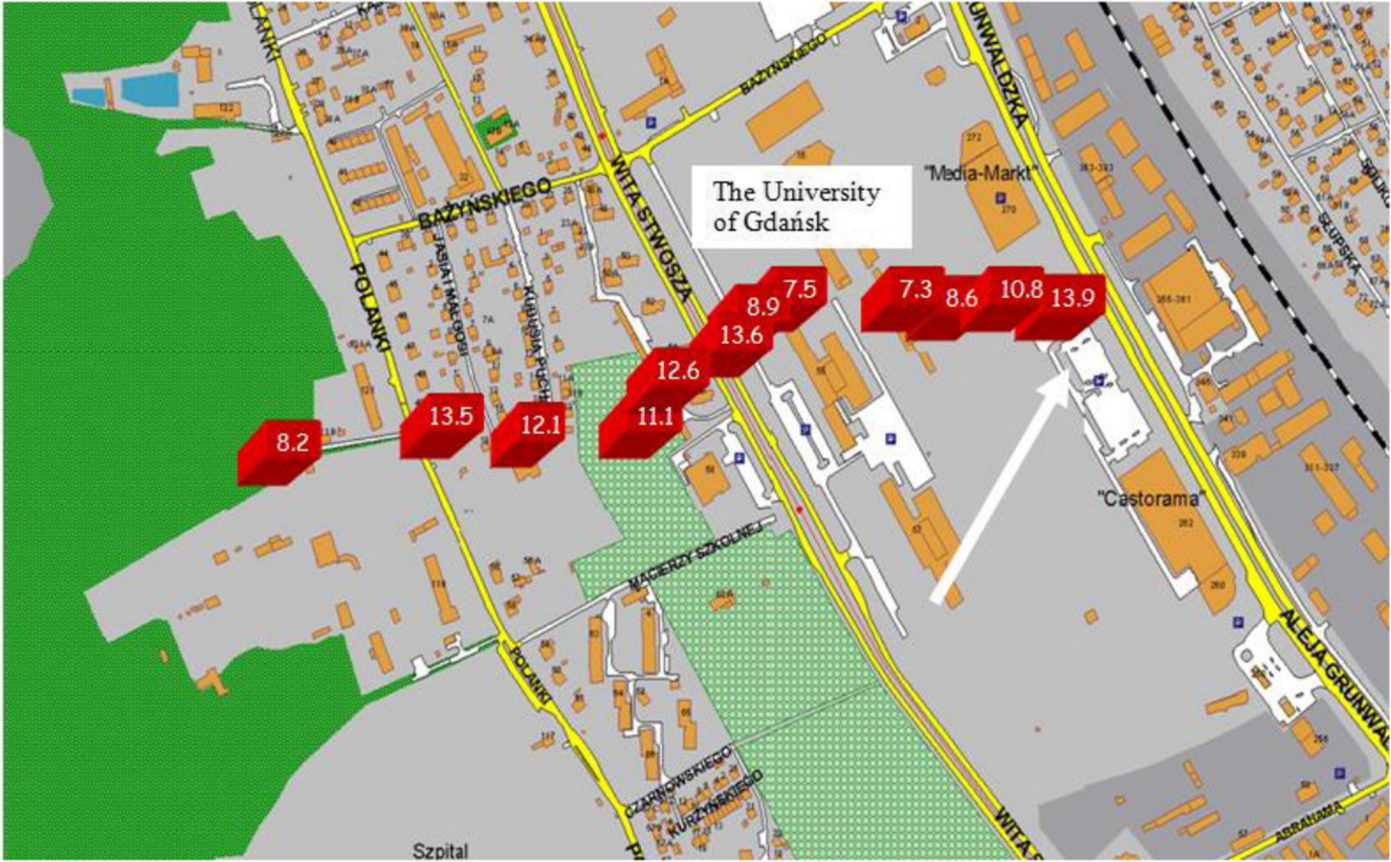
Cross-sectional spatial distribution of elasticity modulus *E*_isoth_ of snowmelt water film recorded in surroundings of University of Gdansk Campus in Oliwa after 3 days elapsed from the snowfall event (on March 7, 2016). The arrow indicates the first sampling place (close to busy street Grunwaldzka). Further collections were continued along a line (perpendicular to the streets) up to the front of the forest wall of the Gdansk Landscape Park. Values of *E*_isoth_ are given in red boxes

A spatial variability of surface active compounds in a snowpack volume can be learned from the cross-sectional profile of *E*_isoth_ distribution taken along a direction perpendicular to the heavy traffic streets area in Gdansk–Oliwa depicted in Fig. 2. The spatial profile of *E*_isoth_ demonstrated the peak values in locations next to heavy traffic roads and at industrial areas remaining under a severe anthropogenic air pollution pressure. The highest values of *E*_isoth_ (= 13.9, 13.6; 13.5 mN m^−1^) were evidenced close to the busy roads, the lowest one (*E*_isoth_ = 7.3 ÷ 7.5 mN m^−1^) appeared for the samples taken far away from motor traffic pressure, and in front of the forest wall (*E*_isoth_ = 8.2 mN m^−1^; Tricity Lanscape Park, Oliwa). There is suggested that certain general classes of components (of highest surface activity) so-called end-members may dominate the snowmelt film surface rheology. It was found that snowpack samples taken in or near urban location show markedly higher polycyclic aromatic hydrocarbons than snow samples taken in rural areas (Sharma and McBean 2001).

**Fig. 3:**
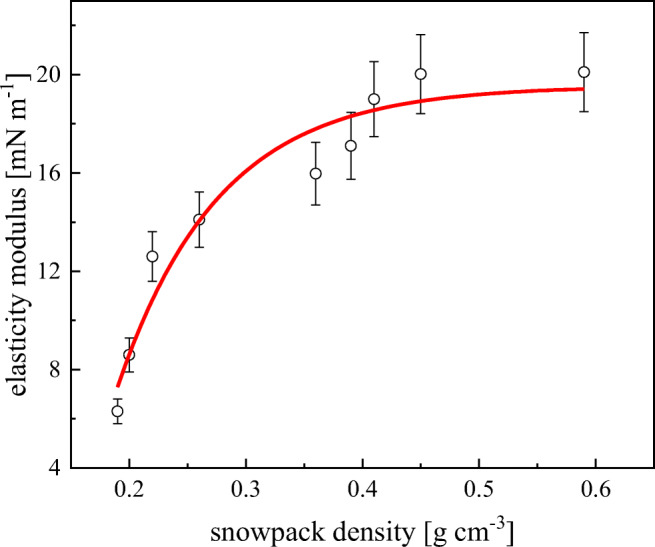
Elasticity modulus *E*_isoth_ of snowmelt water films as a function of snowpack density *ρ*, for samples collected in surroundings of University of Gdansk Campus in Oliwa in the period January 27, to March 9, 2016. The line results from the best-fit procedure applied to the experimental data (Eq. (14))

The snow melting process (an increase of *ρ* ↑) causes incrementally releasing of more surface active organic contaminants capable of forming films with higher values of *E*_isoth_. The film elasticity *E*_isoth_ as a function snow density *ρ* is shown in Fig. 3. There is a close correlation between the film elasticity increase with a snowpack structure evolution from recently fallen snow with a high SSA (1000 cm^2^ g^−1^) of low *ρ* (0.05 g cm^−3^) to aged snow exhibiting low SSA (125 cm^2^ g^−1^) of high *ρ* (0.4 g cm^−3^) related to a large OM concentration (0.18 mg L^−1^) increase in reference, for instance, to Arctic snow (0.009 mg L^−1^; (Wania et al. 1998)).

An exponential power-law relationship between *E*_isoth_ and snow density *ρ* can be derived:

14 $$ {E}_{\mathrm{isoth}}=A{\rho}^B, $$

where *A* = 39.3 ± 0.5 and *B* = 0.9 ± 0.1; *r*^2^ = 0.93 are constants resulting from the best-fit procedure applied to the experimental data.

Assuming that the snowmelt water films are described by the 2D analog of the ideal gas law where Γ = *π*/RT and *π* ~ *E*_isoth_, thus Γ ~ *E*_isoth_/RT. Gibbs adsorption Γ ~ *m*_*f*_/SSA, where *m*_*f*_ is the mass of film-creating material, whereas from a simple geometric consideration, we get SSA ~ *ρ*^3/2^. Putting together all the above dependences, one finally obtains *E*_isoth_/RT ~ *ρ*^2/3^ valid form snowmelt water films of gaseous behavior. As can be noticed, the experimental dependence coefficient *B* in Eq. (14) is equal to 0.9 not 2/3 = 0.67, as foreseen for the simplest film model. Moreover, it is expected that SSA ~ *ρ*^1.11^ rather than ~ *ρ*^1.50 (=3/2)^, for the apparent, real snowpack cover.

### Thermodynamic film characteristics

**Fig. 4:**
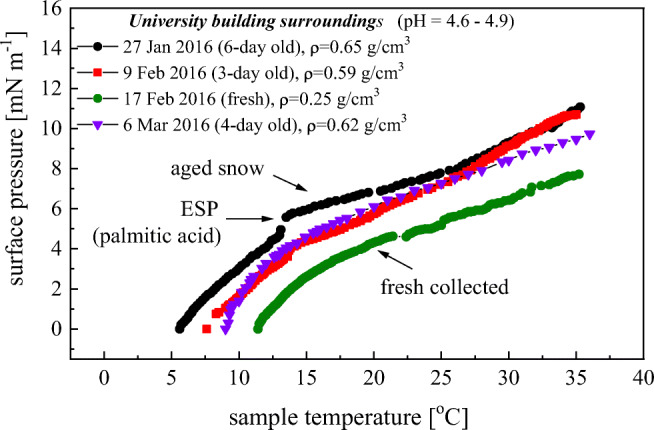
Surface isochors (*π*-*T*)_*A*_ of films on snow-melted water, collected close to (10 m distant) Gdansk University Physics Department building in Oliwa. ESP—equilibrium spreading pressure of palmitic acid a surface active component found in atmospheric waters. Arrows indicate the fresh snowfall sample (lower) and the few-day-old one (upper). In the inset, sampling dates, snow sample age, and snowpack densities are provided

**Fig. 5:**
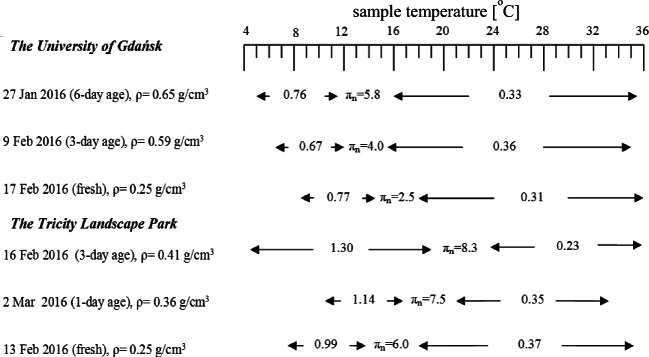
Inflection points (*π*_*n*_, *T*_*n*_) appeared in surface isochors (*π*-*T*)_*A*_ plots, and values of *β*_*s*_ (mN m^−1^ K^−1^), for each linear plot section adjoining the breaking points, determined for snowmelt water samples. Denotations: *π*_*n*_—film surface pressure at the breaking point; arrows indicate the temperature scope of the constant *β*_*s*_ value therein

The isochore plots for surface films formed from snow-melted water, collected at Gdansk are depicted in Fig. 4. The same shape of the isochors can be noticed with the cusp points at *T*_*n*_ = 13 ÷ 17 °C with *π*_*n*_ = 2.5 ÷ 5.8 mN m^−1^, that is correlated with the partial breakdown of stearic acid at its equilibrium surface pressure (ESP), as already evidenced in the isotherms. It also suggests a similar makeup of the film-forming material in snow-derived water. Inflection point positions *T*_*n*_, *π*_*n*_ together with *β*_*s*_ values are summarized in a diagram in Fig. 5. Although, the higher concentration of the surface-active substances in the aged snow (taken after 3, 4, 6 days elapsed from the snowfall event) samples are reflected in the fact that their plots lie over the curve obtained from the fresh snow sampled immediately. Values of *β*_*s*_ for mentioned registrations are almost the same before (0.67 ÷ 0.76) and beyond (0.31 ÷ 0.33) mN m^−1^ K^−1^ the isochore critical point, respectively. In reference to the rain water films (studied in the same location), values of *β*_*s*_ are lower by a factor 2 (Mazurek et al. 2006). Lower *β*_*s*_ values (lower structural entropy) correspond the film structure of less degrees of molecular freedom (more organized) with lower molecule thermal mobility (*π*_*k*_ = *β*_*s*_*T* is lower). Considerably lower *π*_*k*_, ranging from 1.92 ÷ 4.20 (before) to 0.78 ÷ 1.82 mN m^−1^ (beyond) the critical point, were noticed.

A different physicochemical organic material character is observed for snowpack samples taken in a forest area (Tricity Landscape Park). The cusp points are at higher *π*_*n*_ (= 6.0 ÷ 8.3 mN m^−1^) and *T*_*n*_ (= 16 ÷ 22 °C), that can be interpreted as the partial breakdown of palmitic acid at its equilibrium surface pressure (ESP). Values *β*_*s*_ are quite higher (0.99 ÷ 1.30 mN m^−1^ K^−1^) before and similar (0.23 ÷ 0.37 mN m^−1^ K^−1^) beyond the critical point in reference to snowpack samples taken in urban area. That points to less-structured surface films of higher molecules mobility and correlates with the taken *π*_*k*_ changing from 5.84 ÷ 11.3 (before) to 1.95 ÷ 2.69 mN m^−1^ (beyond) at the isochore critical point). Above *T* ≈ 25 °C, values of *β*_*s*_ are similar for natural films of sea surface, inland, and other environmental waters (Mazurek et al. 2008). It is believed that the established variability of rheological surface parameters of snowmelt water films allows one to identify chemical organics makeup to follow the way of snowpack structure evolution and finally to test the existing models on snowfall/ground melting processes.

### Film dilatational viscoelasticity and surface tension gradients

**Fig. 6:**
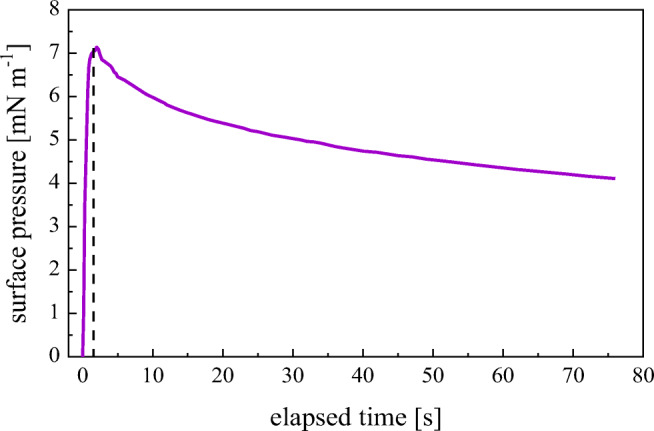
Dynamic surface pressure *π*(*t*) of snowmelt water film (collected at University of Gdansk Campus on March 6, 2016) registered after a step rapid (Δ*t* = 0.8 s) relative area change Δ*A*/*A*_0_ = 0.22; pH = 4.3; *T* = 16 °C. The vertical line points to the step-wise deformation time Δ*t*

An exemplary response of the snowmelt water film to the step, rapid film area compression is depicted in Fig. 6.

First, the principal relaxation times *τ*_*i*_ of the transition processes within the layer were determined from ln *π*(*t*) vs *t* plot (Boniewicz-Szmyt and Pogorzelski 2016), later on the remaining parameters of the surface dilational modulus *E* were derived from Eqs. (10), (11), (12), and (13). The calculated parameter values (mean values and standard deviation (in brackets)) are collected in Table 1.

**Table 1:** Parameter values for an exemplary snowmelt water film calculated in accordance with Eqs. (10), (11), (12), (13). Standard deviations are provided in brackets

**Δ*Α*/*Α***	**Δ*τ*** **(s)**	***τ***_**1**_ **(s)**	***τ***_**2**_ **(s)**	***τ***_**3**_ **(s)**
0.22 (0.01)	1.1 (0.1)	7.21 (0.88)	27.10 (3.29)	62.26 (7.47)
***E***_**isoth**_ **(mN m**^**−1**^**)**	***E***_***d***_ **(mN m**^**−1**^**)**	***E***_***i***_ **(mN m**^**−1**^**)**	**|*E*| (mN m**^**−1**^**)**	***φ*** **(°)**
14.38 (1.74)	8.42 (1.02)	2.90 (0.35)	8.90 (1.05)	19.0 (2.3)

At least three relaxation times of different time scale from 7.21–62.26 s were found for snowmelt water films. The following trend was found *τ* (inland waters) < *τ* (marine waters) < *τ* (rain and snowmelt waters) (based on the data from (Boniewicz-Szmyt and Pogorzelski 2016; Mazurek et al. 2006, 2008)). Several long-lasting processes (molecules aggregation, surfactants separation, partial collapse, etc.) could occur in multicomponent films of atmospheric water samples. Static elasticity modulus *E*_isoth_, determined under thermodynamic equilibrium condition, from (*π*-*A*)_*T*_ isotherms, turned out to be higher by several dozen per cent than modulus |*E*|, in the wide range of surface strain rates ((Δ*A*/*A*_0_)/Δ*t* ≈ 1.15 ÷ 0.18 s^−1^). Moreover, the films exhibited a viscoelastic behavior with a significant account of imaginary part *E*_*i*_ in |*E*| achieving the ratio *E*_*i*_/|*E*| in the range 0.13 ÷ 0.21; much higher ratios were found for rainwater films (0.32 ÷ 0.37). In addition, high values of *φ* (15.6–23.2°) reflected a viscoelastic nature of both class films of atmospheric waters. The modulus of viscoelasticity |*E*| could have been approximated with *E*_isoth_ only for very low strain rates (Δ*A*/*A*_0_)/*dt*. It could be noticed that protein on water film studies revealed a transition of the film character from a purely elastic (*E*_*i*_ ≪ *E*_*d*_, *φ*—low, a few degrees) to viscoelastic (*E*_*i*_ ≈ *E*_*d*_, high, *φ* several dozen degrees), for above the particular value of the strain rate, related to the formation of gelatinous protein molecular complex system (Patino et al. 2003). Mineral dust particles entrapped in a snowpack are capable of forming surface layers exhibiting significant surface pressures and elasticities similarly like natural sea water surfactants (Mazurek and Pogorzelski 2012).

It should be borne in mind that dilatational or compressional deformation of an elastic film-covered water surface will meet opposite resistance force expressed by the corresponding surface tension change:

15 $$ \Delta  {\gamma}_f=E\left(\raisebox{1ex}{$\Delta  A$}\!\left/ \!\raisebox{-1ex}{$A$}\right.\right). $$

So, under dynamic film area change conditions, the effective (lowered) surface tension *γ*_eff_ = *γ*_AW_ − Δ*γ*_*f*_ instead of the equilibrium one has to be taken into account. This effect plays a significant role in any dynamical processes like spreading and retention over liquid or solid substrata, for systems with interfacial elastic films (Boniewicz-Szmyt and Pogorzelski 2018a). For the studied snowmelt water films, Δ*γ*_*f*_ was ranging from 1.25–2.65 mN m^−1^, depending on both the elasticity and area strain.

There is another underestimated surface tension-driven process, which could affect makeup and snowmelt water flow, attributed to the surface tension gradients. The subsurface liquid flow toward surface areas of locally higher surface tension is named Marangoni flow whose horizontal speed *U*_*s*_ depends on the surface tension gradients (Mao et al. 2008). The shear stresses appearing at the interface resulting from surface tension gradients are assumed to be equalized by viscous stresses under hydrodynamic equilibrium conditions. For the two-dimensional coordinate model, where *x* and *z* are horizontal and vertical coordinates, one has (Mao et al. 2008)

16 $$ \mu \frac{\partial {U}_S}{\partial z}=-\frac{\partial \gamma }{\partial T}\frac{\partial T}{\partial x}-\frac{\partial \gamma }{\partial c}\frac{\partial c}{\partial x}, $$

where *U*_*s*_ is the surface flow speed (m s^−1^), *μ* is the liquid viscosity (Pa s), *∂γ*/*∂T* = *γ*_*T*_ is the surface tension temperature coefficient (mN m^−1^ K^−1^), and *∂γ*/*∂c* = *γ*_*c*_ is the surfactant activity (mN m^2^ mol^−1^).

Generally, the apparent surface tension *γ* of a liquid surface with a heterogeneous spatial distribution of surface active substance concentration (Δ*c* = *c* − *c*_0_), and temperature irregularities (Δ*T* = *T* − *T*_0_) reads as

17 $$ \gamma ={\gamma}_0\left[1-{\gamma}_T\left(T-{T}_0\right)-{\gamma}_c\left(c-{c}_0\right)\right]. $$

As it was found in thermo-elastic studies of natural surfactant films in Baltic Sea coastal waters (Boniewicz-Szmyt and Pogorzelski 2018a), the first right-hand term in Eq. (16) related to thermal Marangoni effect took values from the range 52.6–274.2 mN m^−2^, while the second one attributed to classic Marangoni effect achieved only (5.32–10.45 mN m^−2^). They differed by a factor of 10–30, pointing to the principal role played by the temperature over the surfactant-mediated effect. The Marangoni–Benard surface tension gradient-induced cell-like flow in thin liquid layers could be a very effective mixing process leading to redistribution and enrichment of snowpack layer in various surface active organic fraction of dissolved organic matter (DOM). Similar evaluations could be performed for surface active pollutants released from snowpack during the melting process but surfactant concentration and temperature gradients still remain to be determined. Recently, a one-dimensional model for the temporal dynamics of the snowpack has been proposed, accounting for both dry and wet conditions (De Michelle et al. 2013), where a bilinear vertical temperature gradient *∂T*/*∂z* = 0.033 K mm^−1^ was assumed. Having measured here the surface tension temperature coefficients of snowmelt water (*∂γ*/*∂T*), the surface shear stress resulting from the Marangoni flow mechanism (the first right-hand term in Eq. (16)) was evaluated to be 8.2–35.6 mN m^−2^. The obtained values were found to be lower by a factor 10 than measured for natural surfactant layers forming sea surface films (Boniewicz-Szmyt and Pogorzelski 2018a).

## Conclusions

Signatures of the isotherm and isochore dependences of the snowmelt water films revealed that we were concerned with a multicomponent diluted surfactant solution capable of forming G and LE–type films. The presence of slightly soluble long-chain fatty acids (oleic, stearic, palmitic) was exhibited similarly, as already found in our previous surface film studies on rainwater. The snowmelt water films appeared to be interfacial structures with a large degree of molecular architecture heterogeneity (*y* > 10), where several transition processes like surface micelle formation, phase separation of surfactant components, and partial breakdown of differentiated time scales (relaxation times from 7 to 63 s.) took place. The film viscoelasticity modulus components (*E*_*i*_, *E*_*d*_) pointed to the visocoelastic film character (*E*_*i*_/|*E*| ~ 0.13 ÷ 0.21, and *φ* ~ 15.6–23.2°). The film parameters (*E*_isoth_ and *π* in particular) were unequivocally correlated to the snowpack characteristics (*ρ*, SSA), which allowed the functional relations to be proposed, being of practical value for the snowpack melting model testing. In particular, *E*_isoth_ spatial distribution could be an indicator of surface active atmospheric organic contamination accumulation, as revealed in large-scale field studies (surroundings of University of Gdansk Campus at Gdansk-Oliwa). It was postulated that snowmelt water flow during the melting process could be mediated by the film viscoelasticity, which in particular leads to the effective surface tension, instead of the static one, related to the |E| and (Δ*A*/*A*_0_) values of the particular film. Marangoni effect induced by surface tension concentration and temperature gradients, remaining to be estimated in snowpack cover filed studies, leading in turn to an additional snowmelt water flow phenomenon was quantitatively addressed.

## Data Availability

The datasets used and/or analyzed during the current study are available from the corresponding author on reasonable request.
